# Characterization of antibodies induced by immunization of mice with isoglobotrihexosylceramide (iGb3)

**DOI:** 10.1016/j.bbrep.2024.101855

**Published:** 2024-10-30

**Authors:** Tetsuya Okuda

**Affiliations:** Biomedical Research Institute, National Institute of Advanced Industrial Science and Technology (AIST), 1-8-31 Midorigaoka, Ikeda, Osaka, 563-8577, Japan

**Keywords:** α-linked galactose, Isogloboside, Monoclonal antibody, Variable region, IgE

## Abstract

Isoglobotrihexosylceramide (iGb3), a well-characterized natural killer T cell ligand found in mammalian tissues, is also known as a glycosphingolipid that contains the human IgE epitope α-Gal (Galα1,3Gal) structure. Here, we analyzed the reactivity of several mice and human serum immunoglobulins against iGb3. Additionally, we isolated and characterized the variable region sequences of a monoclonal antibody that specifically recognizes iGb3. No IgE reactive with iGb3 was detected in sera from MRL/lpr mice, which are known to produce autoreactive antibodies, or in sera from healthy human donors. Furthermore, no induction of IgE and IgG was observed in the sera of mice immunized with iGb3; only IgM reactivity to iGb3 was detected. Further analysis of an anti-iGb3 monoclonal antibody generated from the splenocytes of an iGb3-immunized mouse revealed that the nucleotide sequences of the variable regions exhibited high homology to those of antibodies recognizing glycoconjugates containing Galα1,3 or Galα1,4 structures. These results indicate that the mouse genome harbors genes capable of encoding antibodies that recognize α-linked galactose-containing glycans, including iGb3, but that iGb3 is not sufficiently immunogenic to induce IgE in mammalian lymphocytes.

## Highlights

• Mouse and human serum does not contain IgE that recognizes iGb3.
• Only IgM reactivity to iGb3 was detected in mice immunized with iGb3.
• A monoclonal IgM that specifically recognizes iGb3 was generated.
• The V_H_ region sequence shows high homology to that of an anti-globoside antibody.
• The V_L_ region sequence shows high homology to that of anti-α-Gal antibodies.

## 1 Introduction

**Fig. 1:**
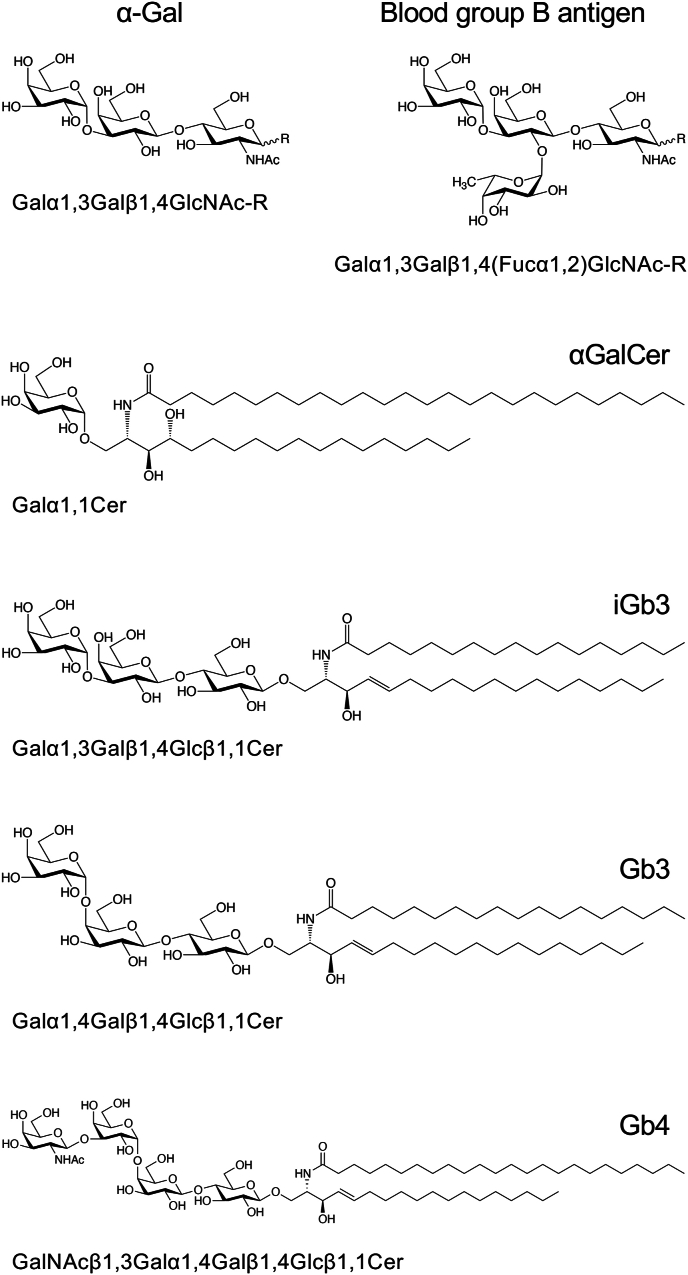
Chemical structures of iGb3 and related glycans. The non-reducing terminal structures of glycoproteins and glycosphingolipids feature α-Gal and the blood group B antigen. As αGalCer, iGb3, Gb3, and Gb4 exhibit a variety of ceramide structures, this figure shows the main ceramide structures of these compounds that were used in this study.

Isoglobotrihexosylceramide (iGb3), a glycosphingolipid component of mammalian cell membranes, has a Galα1,3Gal structure (also termed α-Gal) at its non-reducing end (Fig. 1). iGb3 is known for its role in presenting to human and mouse invariant natural killer T (iNKT) cells by CD1d, which is expressed on antigen-presenting cells and can activate iNKT cells [1,2]. iNKT cells are known to be strongly activated by αGalCer (Fig. 1), a compound found in marine sponges [3], and similar glycolipids derived from microorganisms [2,4]. Although the iNKT cell-activating activity of iGb3 is relatively lower than that of αGalCer, it has been proposed that iGb3 serves as an endogenous ligand for iNKT cells in mammals [1,2]. Conversely, the α-Gal moiety is a carbohydrate antigen that serves as an epitope for human IgE [5,6]. Although α-Gal is structurally similar to the human blood group B antigen (Fig. 1), it has not been found in human glycoproteins and glycolipids [7]. Although present in edible animals [8] and rodent-derived cells used antibody drug production [5], glycoproteins and glycolipids derived from these sources may trigger allergic reactions such as anaphylaxis in anti-α-Gal IgE carriers [5,6,8].

While the role of iGb3 as a ligand for iNKT cells has been studied extensively, its potential as an IgE epitope remains largely unknown. To investigate whether iGb3 exhibits IgE-inducing activity, we investigated IgE induction in serum samples from autoimmune disease model MRL/lpr mice, healthy human donors, and mice immunized with iGb3. No IgE reactivity with iGb3 was detected in these serum samples, but IgM reactivity was induced in the sera of mice immunized with iGb3. Further analysis of an iGb3-specific monoclonal antibody generated from an iGb3-immunized mouse revealed that the variable region sequence of this antibody showed high homology to those of antibodies that recognize α-Gal or α1,4-linked galactose-containing glycosphingolipids (Fig. 1). These findings suggest that while the mouse genome encodes genes for constructing antibodies that recognize α-linked galactose-containing glycans including iGb3, iGb3 by itself is not sufficiently immunogenic to induce IgE in mammalian lymphocytes.

## 2 Materials and methods

### 2.1 Materials

iGb3 and other carbohydrate antigens were obtained from Sigma-Aldrich (St. Louis, MO, USA), or prepared as described previously [9]. Sp2/0-Ag14 myeloma cells, MRMT-1 rat breast carcinoma cells, and HeLa cells were obtained from Riken Cell Bank (Tsukuba, Japan). Human serum obtained from a variety of blood types and different sexes was obtained from Biowest (Nuaillé, France); this human serum was collected or imported and treated in accordance with European regulations. Horseradish peroxidase (HRP)-labeled goat anti-mouse IgM and anti-mouse IgG were obtained from Sigma-Aldrich. HRP-labeled goat anti-mouse IgE and anti-human IgE were obtained from Bethyl Laboratories (Montgomery, Texas, USA) and Thermo Fisher Scientific (Waltham, MA, USA), respectively.

### 2.2 Immunization and preparation of serum

Female C3H/HeN mice (CLEA Japan, Tokyo) were immunized with iGb3 using a liposome immunization method [10,11]. Briefly, 100 μg of iGb3 was mixed with 10 μg of lipid A, 0.5 μmol of cholesterol, and 0.5 μmol of dipalmitoylphosphatidylcholine. The mixture was then dissolved in phosphate-buffered saline (PBS) and used as an immunogen. Mice were initially immunized subcutaneously and then intraperitoneally at 2 weeks after the first immunization. Serum was prepared from cardiac blood collected 3 days after the second immunization, and spleens were also harvested for the generation of hybridoma cells. Lupus model MRL/lpr mice were obtained from Japan SLC (Shizuoka, Japan). Whole blood was collected from the heart of female MRL/lpr mice at 7–8 weeks of age, and the blood samples from 10 mice were pooled. After incubating the collected blood at 37 °C for 90 min, the clots were removed, and the sample was centrifuged at 3000 rpm for 15 min to collect the supernatant as serum.

All mice were housed in a controlled specific pathogen-free animal room at a temperature of 23 °C ± 2 °C and a humidity of 55 % ± 15 % under a 12 h light/12 h dark cycle. The animals were given access to food and water ad libitum. All of the animal experiments were approved by the Committee for Experiments Involving Animals of the National Institute of Advanced Industrial Science and Technology (approval number: 0119) and were performed in accordance with the relevant guidelines and regulations. Findings were reported in accordance with ARRIVE guidelines 28.

### 2.3 Enzyme-linked immunosorbent assay (ELISA)

ELISA analysis was performed as described previously [9]. For glycosphingolipids, 500 ng of sample dissolved in methanol was added to each well of a 96-well microtiter plate, then incubated and allowed to dry for fixation onto the plate. For glycoproteins, 1 μg of sample dissolved in PBS was applied to each well of the plate, which was incubated overnight at 4 °C to promote fixation onto the plate and then washed twice with PBS. For single-stranded DNA (ssDNA) and double-stranded DNA (dsDNA) assays, the wells of a 96-well microtiter plate were pre-coated with a 0.0125 % ε-Poly-l-lysine solution (COSMO BIO, Tokyo, Japan). Subsequently, 250 ng of either ssDNA or dsDNA dissolved in distilled water was added to each well to facilitate fixation of the DNA onto the plate. Blocking buffer (1 % BSA in PBS) was then added to the antigen-coated wells, and incubated for 15 min at room temperature, followed by the addition of diluted antibodies. After incubation for 2.5 h at room temperature, the wells were washed with 0.05 % Tween 20 in PBS, then HRP-labeled secondary antibodies were added. Binding of HRP-labeled secondary antibody to the primary antibody was detected using an HRP substrate (1-Step Ultra TMB-ELISA Substrate; Thermo Fisher Scientific), and the absorbance was measured at 450 nm. Samples were analyzed in duplicate in each experiment. Serum IgE levels were quantified using the LBIS Mouse IgE ELISA Kit (Wako, Osaka, Japan).

### 2.4 Hybridoma generation and characterization of immunoglobulin isotypes

Splenocytes collected from an iGb3-immunized mouse were fused with mouse Sp2/0-Ag14 myeloma cells as described previously [10]. The fused cells were then seeded into 96-well microtiter plates to grow as a single colony per well, and hybridomas were selected using a hypoxanthine-aminopterin-thymidine selection medium (RPMI-1640 containing 10 % fetal calf serum, 0.1 mM sodium hypoxanthine, 0.4 μM aminopterin, 16 μM thymidine, 10 μg/ml gentamicin, and 5 % Briclone [DS Pharma Biomedical, Osaka, Japan]). Cells were cultured at 37 °C in a humidified atmosphere containing 5 % CO_2_. Culture supernatants were evaluated by ELISA, and clones showing positive reactivity against iGb3 were selected. The amount of IgM in the culture supernatant was measured using an IgM ELISA kit (Thermo Fisher Scientific). The isotype of monoclonal antibody was determined using a mouse monoclonal antibody isotyping kit (Roche Diagnostics GmbH, Mannheim, Germany).

### 2.5 Flow cytometric analysis

The expression of iGb3 on the cell surface was analyzed using an RF-500 flow cytometer (Sysmex, Tokyo, Japan). MRMT-1 and HeLa cells were maintained on culture dishes (100 mm diameter) in RPMI-1640 medium containing 10 % fetal bovine serum, 100 units/ml penicillin, and 100 μg/ml streptomycin. Cells were harvested after overnight culture (37 °C, 5 % CO_2_), and 1 × 10^6^ cells were suspended in 200 μl of cold PBS. To detect cell surface glycan antigens, the suspensions were incubated on ice with 1 μg of 2D1 monoclonal IgM, followed by sequential labeling with Alexa 488-conjugated anti-mouse IgM antibody (Thermo Fisher Scientific).

### 2.6 Determination of the nucleotide sequences of immunoglobulin variable region genes

Determination of the nucleotide sequences of the immunoglobulin variable region genes was outsourced to Bio-Peak (Gunma, Japan) and was performed as described previously [12]. The genes encoding the heavy chain variable region (V_H_) and light chain variable region (V_L_) were amplified by polymerase chain reaction using degenerate primers and cDNA synthesized from total RNA isolated from hybridoma cells. The nucleotide sequences of the V_H_ and V_L_ variable region genes were determined by sequencing the amplified DNA, and the complementarity determining regions (CDRs) in the V_H_ and V_L_ genes were identified using the Kabat numbering scheme. Homology searches of target gene sequences against core nucleotide database and mouse genomic DNA database were performed using the blastn program of BLAST (https://blast.ncbi.nlm.nih.gov/Blast.cgi). Homology analysis of nucleotide and amino acid sequences of target genes was performed using GENETYX-MAC (GENETYX, Tokyo, Japan). The nucleotide sequences reported in this paper were submitted to GenBank under accession numbers LC829500 (V_H_ of 2D1), LC829501 (V_L_ of 2D1), LC730315 (V_H_ of PA4.2), and LC730316 (V_L_ of PA4.2).

## 3 Results & discussion

### 3.1 Analysis of serum immunoglobulins that recognize iGb3

**Fig. 2:**
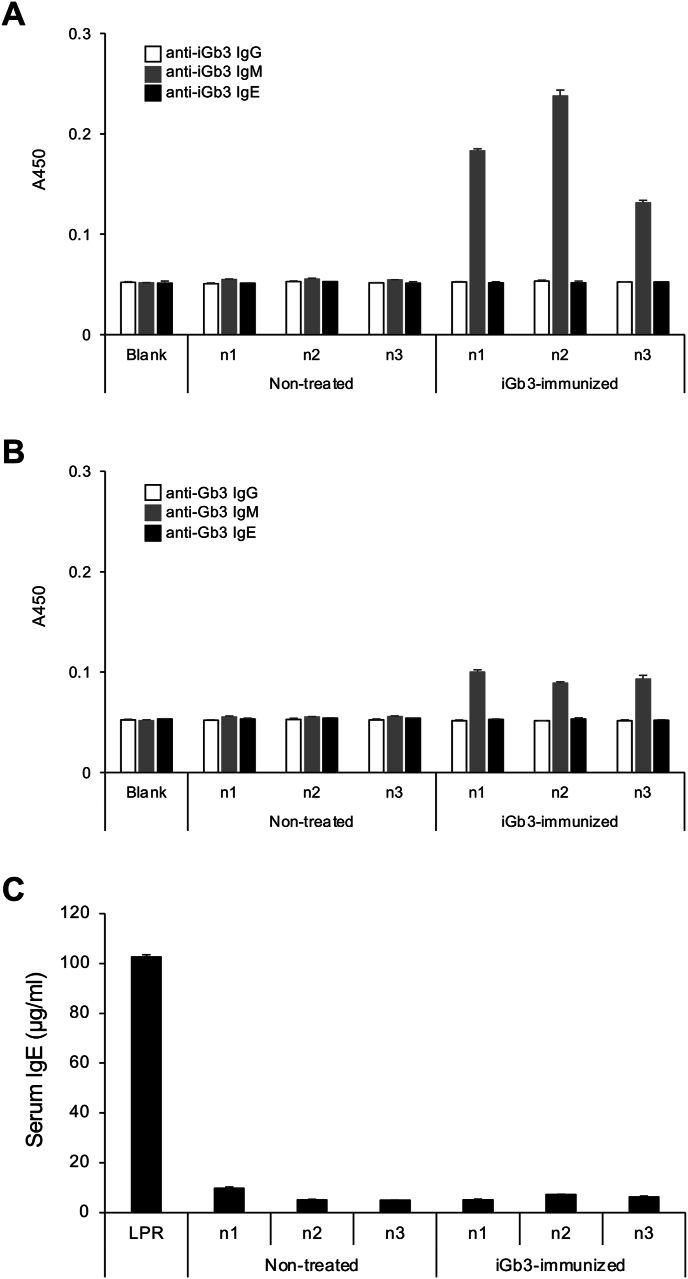
ELISA analysis of anti-iGb3 immunoglobulins in sera from mice immunized with iGb3. (A and B) Mice were serially immunized with iGb3-containing liposomes as described in the Materials and Methods. Serum samples were collected at 3 days after the final immunization. The reactivity of IgG (white bars), IgM (gray bars), and IgE (black bars) in serum to iGb3 (A) or Gb3 (B) was analyzed by ELISA. Results for three mice (n1, n2, n3) are presented, with serum from untreated mouse serving as baseline controls. Error bars indicate the mean ± standard deviation. ELISA results obtained using only the secondary detection antibody are shown as negative controls (Blank). (C) Total IgE levels (μg/ml) in the serum of iGb3-immunized mice. The total IgE level in pooled serum prepared from MRL/lpr mice (LPR) is shown as a positive control.

In rodents, iGb3 is expressed in a variety of rat tissues [13,14], but is barely expressed in mice tissues, except in limited areas such as the dorsal root ganglion [15]. Thus, we hypothesized that iGb3 might be antigenic in mice and that endogenous anti-iGb3 antibodies could be present in the serum. To test these hypotheses, we analyzed the reactivity of IgG, IgM, and IgE serum fractions from mice to iGb3 using ELISA (Fig. 2). Contrary to expectations, none of the immunoglobulin fractions in the serum of non-treated mice, nor the IgG and IgE fractions of iGb3-immunized mice, showed reactivity to iGb3. Reactivity to iGb3 was observed only in the IgM fraction of sera from mice immunized with iGb3 (Fig. 2A). Furthermore, these IgMs also showed slight reactivity to Gb3 (Fig. 1, Gb3), a glycolipid that is structurally similar to iGb3 (Fig. 2B). Thus, to verify that the sera of iGb3-immunized mice contained IgMs that specifically recognized iGb3, it was necessary to isolate and analyze monoclonal IgMs.

We also analyzed whether immunization with iGb3 increased total IgE production in the serum of these mice (Fig. 2C). However, no significant increase in IgE was detected between non-treated and iGb3-immunized mice. In this analysis, a pooled serum prepared from MRL/lpr mice, a type of model mouse for the autoimmune disease lupus erythematosus [16], was used as a positive control (Fig. 2C, LPR). Since MRL/lpr mice are known to produce autoreactive antibodies and considering that their serum in this study contained large amounts of IgE, we also analyzed whether this serum contained IgE reactive with iGb3 or other carbohydrate antigens. However, ELISA analysis of various carbohydrate antigens, including iGb3, revealed no reactivity of the serum IgE to these antigens (Supplementary Fig. S1A). We also assessed the reactivity of IgE in pooled serum from healthy human donors against these carbohydrate antigens, and similarly found no detectable reactivity (Supplementary Fig. S1B).

Stimulation of iNKT cells with αGalCer promotes the production of cytokines that induce antibody class switching to IgE. Thus, the use of αGalCer as an adjuvant to induce IgE production has also been investigated [17,18]. Although we expected iGb3 to have a similar adjuvant effect, no induction of IgE was detected in mice immunized with iGb3. Unlike αGalCer, iGb3 does not appear to be an effective adjuvant for inducing IgE production in mice. This result indicates that iGb3 has limited immunological properties compared to other iNKT cell ligand glycolipids.

### 3.2 Analysis of a monoclonal IgM isolated from an iGb3-immunized mouse

**Fig. 3:**
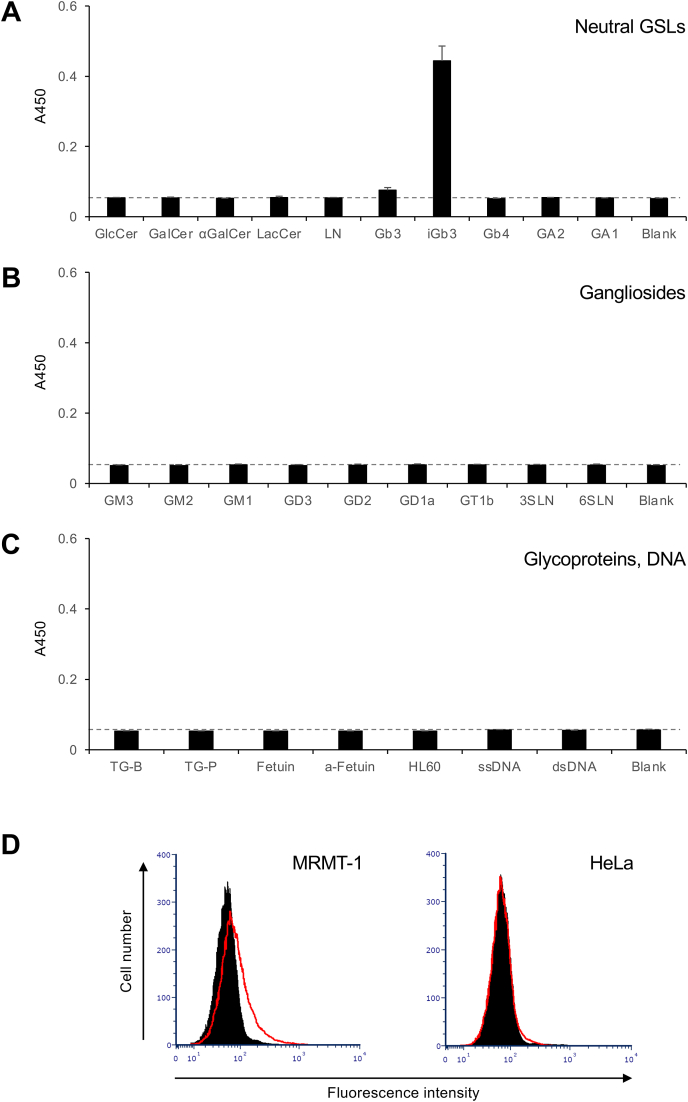
Reactivity of the 2D1 monoclonal IgM antibody to various carbohydrate antigens. (A–C) Microtiter plate wells coated with various neutral glycosphingolipids (A), gangliosides (B), and glycoproteins and DNA (C) were incubated with a dilute solution of 2D1 monoclonal IgM (5 μg/ml). The reactivities of this IgM to the antigens in each well were detected by ELISA. Error bars indicate the mean ± standard deviation (n = 2). Abbreviations: TG-B, bovine thyroglobulin; TG-P, porcine thyroglobulin; a-Fetuin, asialo-fetuin; HL60, glycoproteins extracted from HL60 cells; ssDNA, single-stranded DNA; dsDNA, double-stranded DNA. The chemical structures of the glycosphingolipids used in this analysis are shown in Supplementary Table S1. Thyroglobulins contain glycans with α-Gal as the terminal structure [19]. Fetuin is a glycoprotein that contains primarily sialic acid at the terminus of *N*-linked glycans [29]. Asialo-fetuin is prepared by removing the terminal sialic acid from the glycans of Fetuin, so that Galβ1,4GlcNAc becomes the predominant terminal structure of the glycans [10]. The glycoproteins extracted from HL60 cells contain adhesive glycans such as sialy-Lewis^X^ [11], which are prevalent in immune cells. (D) Flow cytometric analysis of 2D1 monoclonal IgM reactivity to cell surface antigens. MRMT-1 (left panel) and HeLa (right panel) cells were incubated with 5 μg/ml of 2D1 IgM as the primary antibody, followed by an Alexa 488-labeled secondary antibody (red lines). Controls for background monitoring were prepared using a standard mouse IgM and the secondary antibody (dark shading).

To confirm that the serum of iGb3-immunized mice contained IgM that specifically recognized iGb3, we generated hybridomas from an iGb3-immunized mouse and analyzed the reactivity of the monoclonal antibodies produced by these hybridomas to iGb3. Hybridomas were generated using splenocytes isolated from a mouse in which the serum exhibited strong reactivity to iGb3 among the immunized mice. We screened 384 single hybridoma colonies and selected seven hybridoma clones that produced immunoglobulins that strongly reacted with iGb3. We then analyzed the reactivity of these immunoglobulins to Gb3 and isolated three hybridoma clones that produced immunoglobulins that reacted with iGb3 but not with Gb3. All of these immunoglobulins were IgM(κ). Although we expected that some anti-iGb3 monoclonal antibodies would cross-react with α-Gal-containing glycoproteins (bovine and porcine thyroglobulin) [19], no such cross-reactivity was observed with any of the monoclonal antibodies tested. Among the three isolated hybridomas, we established hybridoma clone 2D1, which produced IgM demonstrating the strongest reactivity with iGb3 compared to other IgMs. ELISA analysis of its reactivity to various carbohydrate antigens showed that 2D1 IgM reacts specifically with iGb3, and shows no reactivity with other neutral glycosphingolipids, gangliosides, glycoproteins, or DNA (Fig. 3A–C). Additionally, flow cytometry analysis confirmed that 2D1 IgM reacted with rat MRMT-1 cells, which express iGb3 [20], but not with human-derived HeLa cells, which do not express iGb3 (Fig. 3D).

### 3.3 Nucleotide sequences of the variable regions of anti-iGb3 IgM

**Fig. 4:**
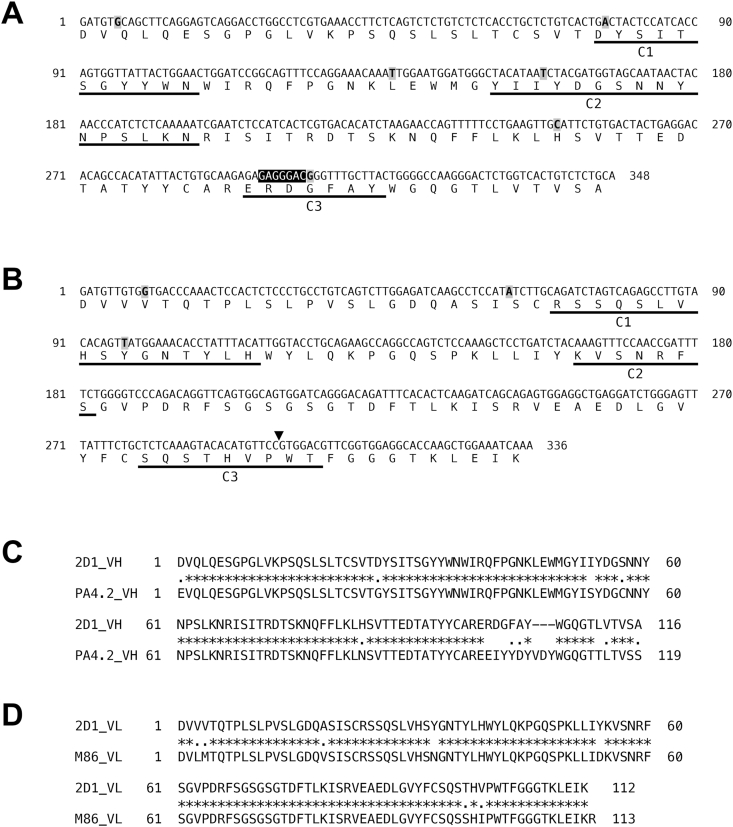
Nucleotide and amino acid sequences of the variable regions of the 2D1 monoclonal antibody. (A) V_H_ gene segment sequences and inferred amino acid sequences of the 2D1 anti-iGb3 monoclonal IgM. The D gene segment is highlighted by a closed box, flanked upstream by the V segment and downstream by the J segment. (B) V_L_ gene segment sequences and inferred amino acid sequences of 2D1. The arrowhead indicates the division between the upstream V segment and the downstream J segment. Bases that differ from the mouse genomic V_H_ and V_L_ sequences are highlighted by gray boxes. Complementarity determining regions (CDRs, C1, C2, C3) are underlined. (C) Homology analysis of V_H_ amino acid sequences between 2D1 and the anti-Gb4 PA4.2 antibody. (D) Homology analysis of V_L_ amino acid sequences between 2D1 and the anti-α-Gal M86 antibody. Asterisks and dots indicate conserved amino acids and amino acids with similar properties, respectively.

To better understand the properties of the anti-iGb3 antibody, we analyzed the nucleotide sequences of the variable regions of the 2D1 IgM. cDNA prepared from total RNA isolated from the 2D1 hybridomas was used to determine the nucleotide sequences. BLAST searches against public databases revealed that the V_H_ region of 2D1 is composed of the Ighv3-6 (V segment), Ighd3-3 (D segment), and Ighj3 (J segment) gene segments (Fig. 4A), while the V_L_ region consisted of the Igkv1-110 (V segment) and Igkj1 (J segment) gene segments (Fig. 4B). Compared to the mouse genomic DNA sequence, some mutations were found in the sequences of both the V_H_ and V_L_ regions (Fig. 4A and B, highlighted gray). The results of further homology searches against genes deposited in public databases revealed that these V_H_ and V_L_ gene regions showed high homology with an anti-Gb4 IgG3 (clone ID: PA4.2) and anti-α-Gal IgMs (clone IDs: M86 and B4H2), respectively. The PA4.2 IgG3, a monoclonal antibody established by our group [12,21,22], specifically reacts with Gb4/globoside, a glycosphingolipid that contains a α1,4-linked galactose (Fig. 1, Gb4). The M86 and B4H2 IgMs, established using α1,3-galactosyltransferase knockout mice that lack the α-Gal epitopes in their tissues, are reactive towards α-Gal-containing glycoproteins and glycolipids [23]. The nucleotide sequence of the V_H_ region of the 2D1 IgM exhibited 90 % homology with that of PA4.2 IgG3 (Fig. 4C), whereas the V_L_ regions exhibited a lower similarity (70 %). In contrast, the V_H_ region of 2D1 showed only 70 % similarity with that of M86 and B4H2 IgMs, while the V_L_ regions were highly conserved, exhibiting 96 % similarity. The amino acid sequence homologies of the V_H_ regions of 2D1 and PA4.2, and the V_L_ regions of 2D1 and M86/B4H2 IgMs were 86 % and 93 %, respectively. Since the V_L_ amino acid sequences of M86 and B4H2 IgM are identical, only the sequence of M86 is shown in Fig. 4D. These findings suggest that the V_H_ region of 2D1 is involved in recognizing a common structural motif between iGb3 and Gb4, whereas the V_L_ region is involved in recognizing the terminal Galα1,3Gal structure.

Mutations in the nucleotide sequences of the 2D1 V_H_ and V_L_ regions were accompanied by amino acid substitutions, particularly in the CDRs, where complete substitutions were observed. Compared to the translation from the genomic sequence, the CDRs of the V_H_ region exhibited three amino acid substitutions (G26D, S53I, and Y102G) that were absent in the V_H_ region of PA4.2 (Fig. 4A and C). Additionally, the CDRs of the V_L_ region contained one amino acid substitution in the C2 domain (N33Y) that was not observed in the V_L_ region of M86/B4H2 (Fig. 4B and D). These results suggest that the production of antibodies that specifically recognize iGb3 is dependent upon amino acid substitutions in the CDRs in conjunction with robust immune induction and somatic hypermutation. However, analyses of various sera failed to detect anti-iGb3 IgE and IgG, and even immunization with iGb3 did not induce class switching in anti-iGb3 antibodies. Thus, iGb3 appears to be insufficiently antigenic to induce IgE production in mammalian immune cells.

Based on these findings, we conclude that iGb3 is unlikely to act as an IgE-inducing antigen in mice. Some of the antibodies induced in iGb3-immunized mice cross-react with Gb3 (Fig. 2B), and if IgE reactive to iGb3 are induced, these may also react to Gb3. Since Gb3 is abundant in mouse tissues [24], such IgE is thought to cause severe allergic reactions in mice. Thus, the mouse immune system may be regulated to prevent the production of IgE that recognizes iGb3. iGb3 would exhibit immunological properties in humans similar to those in mice because Gb3 is also abundant in human tissues [25]. Our findings suggest that iGb3 is a glycolipid antigen that can promote iNKT cell-mediated immune regulation without inducing allergic responses. The primary distinction between the human IgE epitope α-Gal and iGb3 lies in the saccharide at the reducing end, i.e., GlcNAc in α-Gal or glucose in iGb3. We propose that the N-acetylation at the 2nd position of glucose plays a role in the IgE-inducing activity of α-Gal. On the other hand, to the best of our knowledge, 2D1 IgM is the first antibody that specifically recognizes iGb3. Although several antibodies that recognize iGb3 have been reported [[26], [27], [28]], these antibodies also exhibit cross-reactivity with α-Gal or Gb3. This cross-reactivity complicates the interpretation of immune responses to each α-linked galactose containing glycans. The successful isolation of the iGb3-specific 2D1 antibody is expected to accelerate further research into the biological functions of iGb3 and its potential applications.

## CRediT authorship contribution statement

**Tetsuya Okuda:** Writing – review & editing, Writing – original draft, Validation, Resources, Project administration, Methodology, Investigation, Funding acquisition, Formal analysis, Data curation, Conceptualization.

## Declaration of competing interest

The authors declare the following financial interests/personal relationships which may be considered as potential competing interests:

Tetsuya Okuda reports financial support was provided by 10.13039/501100001691Japan Society for the Promotion of Science. If there are other authors, they declare that they have no known competing financial interests or personal relationships that could have appeared to influence the work reported in this paper.

## Data Availability

Data will be made available on request.
