# Supplementary material for: Characterization of antibodies induced by immunization of mice with isoglobotrihexosylceramide (iGb3)
Source: Biochem Biophys Rep. 2024 Oct 30;40:101855. doi: 10.1016/j.bbrep.2024.101855 (PMC11564984; doi:10.1016/j.bbrep.2024.101855)
Supplement: Multimedia component 1 [file mmc1.pptx]

## Slide 1
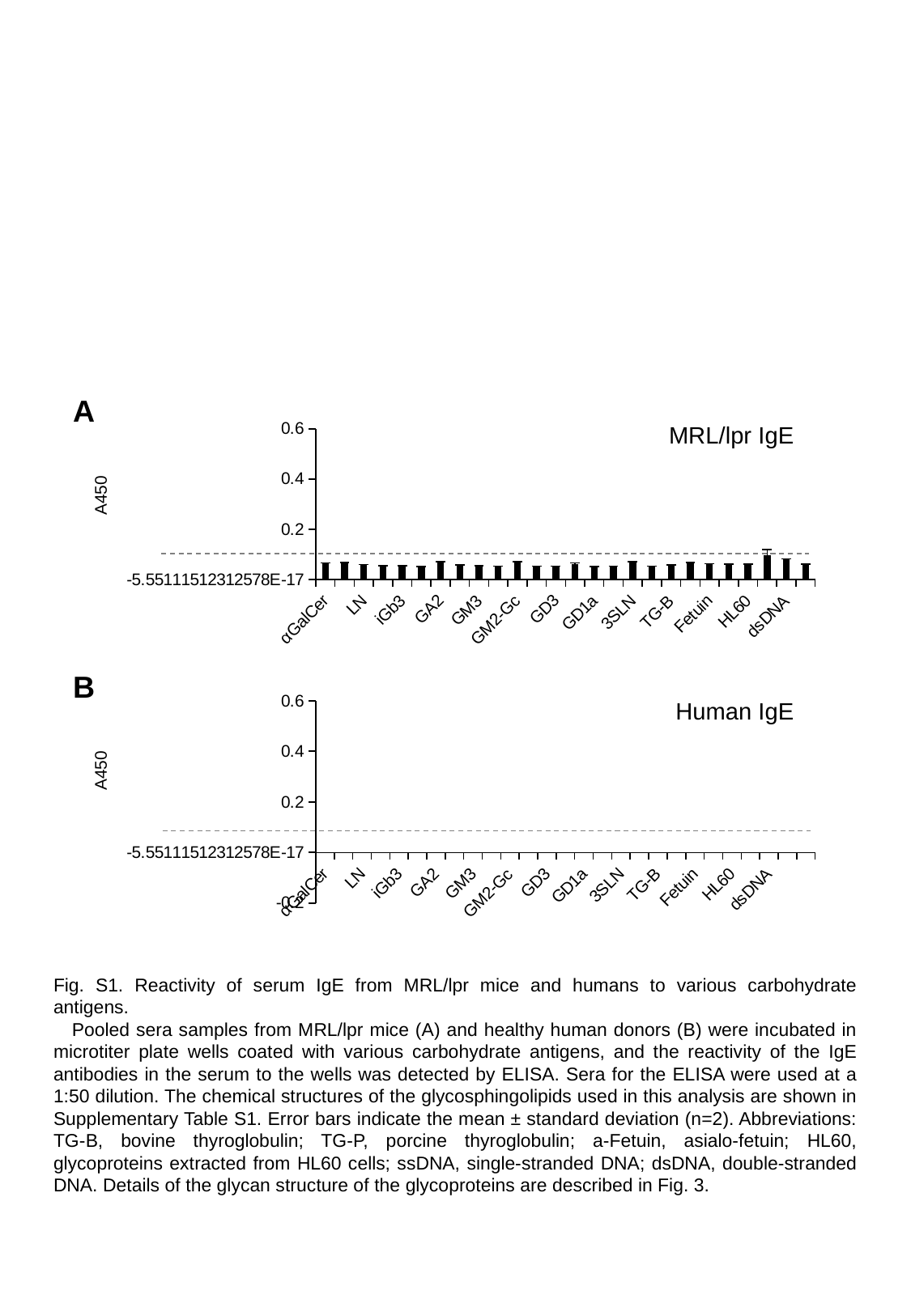

A
### Chart
| Category | A450 |
|---|---|
| αGalCer | 0.06356666666666666 |
| LacCer | 0.06525 |
| LN | 0.057800000000000004 |
| Gb3 | 0.05245 |
| iGb3 | 0.0538 |
| Gb4 | 0.05155 |
| GA2 | 0.0672 |
| GA1 | 0.05815 |
| GM3 | 0.05425 |
| GM2 | 0.05245 |
| GM2-Gc | 0.06603333333333335 |
| GM1 | 0.05265 |
| GD3 | 0.052849999999999994 |
| GD2 | 0.06066666666666667 |
| GD1a | 0.051199999999999996 |
| GT1b | 0.05235 |
| 3SLN | 0.06805 |
| 6SLN | 0.05285 |
| TG-B | 0.058550000000000005 |
| TG-P | 0.0653 |
| Fetuin | 0.06015 |
| a-Fetuin | 0.060649999999999996 |
| HL60 | 0.05925 |
| ssDNA | 0.0943 |
| dsDNA | 0.079725 |
| Blank | 0.05910000000000001 |MRL/lpr IgE
A450
B
### Chart
| Category | A450 |
|---|---|
| αGalCer | 0.07383333333333332 |
| LacCer | 0.061 |
| LN | 0.05925 |
| Gb3 | 0.0555 |
| iGb3 | 0.0553 |
| Gb4 | 0.05215 |
| GA2 | 0.06055 |
| GA1 | 0.05875 |
| GM3 | 0.0651 |
| GM2 | 0.055 |
| GM2-Gc | 0.06135 |
| GM1 | 0.06815 |
| GD3 | 0.055150000000000005 |
| GD2 | 0.05439999999999999 |
| GD1a | 0.05675 |
| GT1b | 0.055650000000000005 |
| 3SLN | 0.0673 |
| 6SLN | 0.055099999999999996 |
| TG-B | 0.0658 |
| TG-P | 0.07202499999999999 |
| Fetuin | 0.057050000000000003 |
| a-Fetuin | 0.0572 |
| HL60 | 0.0581 |
| ssDNA | 0.078425 |
| dsDNA | 0.07529999999999999 |
| Blank | 0.057475 |Human IgE
A450
Fig. S1. Reactivity of serum IgE from MRL/lpr mice and humans to various carbohydrate antigens.
Pooled sera samples from MRL/lpr mice (A) and healthy human donors (B) were incubated in microtiter plate wells coated with various carbohydrate antigens, and the reactivity of the IgE antibodies in the serum to the wells was detected by ELISA. Sera for the ELISA were used at a 1:50 dilution. The chemical structures of the glycosphingolipids used in this analysis are shown in Supplementary Table S1. Error bars indicate the mean ± standard deviation (n=2). Abbreviations: TG-B, bovine thyroglobulin; TG-P, porcine thyroglobulin; a-Fetuin, asialo-fetuin; HL60, glycoproteins extracted from HL60 cells; ssDNA, single-stranded DNA; dsDNA, double-stranded DNA. Details of the glycan structure of the glycoproteins are described in Fig. 3.
