# Supplementary material for: Characterization of antibodies induced by immunization of mice with isoglobotrihexosylceramide (iGb3)
Source: Biochem Biophys Rep. 2024 Oct 30;40:101855. doi: 10.1016/j.bbrep.2024.101855 (PMC11564984; doi:10.1016/j.bbrep.2024.101855)
Supplement: Multimedia component 2 [file mmc2.docx]

Abbreviation Structure

GlcCer Glcβ1,1Cer

GalCer Galβ1,1Cer

αGalCer Galα1,1Cer

LacCer Galβ1,4Glcβ1,1Cer

LN Galβ1,4GlcNAcβ1,1CerA

Gb3 Galα1,4Galβ1,4Glcβ1,1Cer

iGb3 Galα1,3Galβ1,4Glcβ1,1Cer

Gb4 GalNAcβ1,3Galα1,4Galβ1,4Glcβ1,1Cer

GA2 GalNAcβ1,4Galβ1,4Glcβ1,1Cer

GA1 Galβ1,3GalNAcβ1,4Galβ1,4Glcβ1,1Cer

GM3 Siaα2,3Galβ1,4Glcβ1,1Cer

GM2 GalNAcβ1,4(Siaα2,3)Galβ1,4Glcβ1,1Cer

GM1 Galβ1,3GalNAcβ1,4(Siaα2,3)Galβ1,4Glcβ1,1Cer

GD3 Siaα2,8Siaα2,3Galβ1,4Glcβ1,1Cer

GD2 GalNAcβ1,4(Siaα2,8Siaα2,3)Galβ1,4Glcβ1,1Cer

GD1a Siaα2,3Galβ1,3GalNAcβ1,4(Siaα2,3)Galβ1,4Glcβ1,1Cer

GT1b Siaα2,3Galβ1,3GalNAcβ1,4(Siaα2,8Siaα2,3)Galβ1,4Glcβ1,1Cer

3SLN Siaα2,3Galβ1,4GlcNAcβ1,1CerA

6SLN Siaα2,6Galβ1,4GlcNAcβ1,1CerA

Table S1. Chemical structures of the glycosphingolipids used in the ELISA analysis.

Abbreviations: Glc, glucose; Cer, ceramide; CerA, ceramide analogue; Gal, galactose; Gb4, Gb4Cer; GalNAc, *N*-acetylgalactosamine; Sia, sialic acid (Neu5Ac).
